# The surgery for the patients with intestinal non‑Hodgkin lymphomas: a nationwide study

**DOI:** 10.1080/07853890.2026.2634447

**Published:** 2026-02-24

**Authors:** Jiyeong Kim, Jun Ho Lee, Sung Hwan Hwang, Jung Hye Choi, Young-Woong Won, Joon Young Hur

**Affiliations:** a Department of Pre-Medicine, College of Medicine, and Biostatistics Laboratory, Medical Research Collaborating Center, Hanyang University, Seoul, Republic of Korea; b Department of Surgery, Hanyang University Guri Hospital, Hanyang University College of Medicine, Guri, Gyeonggi-do, Republic of Korea; c Division of Hematology and Oncology, Department of Internal Medicine, Hanyang University Guri Hospital, Hanyang University College of Medicine, Guri, Gyeonggi-do, Republic of Korea; d Department of Hematology, Asan Medical Center, University of Ulsan College of Medicine, Seoul, Republic of Korea

**Keywords:** intestinal, lymphoma, surgery, survival

## Abstract

**Background:**

The treatment strategy for intestinal non-Hodgkin lymphoma (NHL) and the role of surgery warrant reevaluation.

**Methods:**

This study analyzed clinical data from a cohort of 12,047 patients diagnosed with intestinal NHL, extracted from the Korean National Health Insurance System database between 2002 and 2021.

**Results:**

Among these patients, 3,566 (29.6%) were categorized into the surgery group, while 8,481 (70.4%) were included in the nonsurgery group. Surgery was independently associated with both prolonged overall survival (OS) and a favorable prognosis in multivariate analysis (Hazard Ratio [HR] = 0.645, 95% Confidence Interval [CI] = 0.598–0.695, *p* <.001). The median OS was longer in patients who underwent lymph node dissection during surgery than in patients who did not undergo lymph node dissection (10-year OS with lymph node dissection 63.17% vs. surgery without lymph node dissection 54.78%, *p* < .001).

**Conclusions:**

To our knowledge, this is the first Korean population-based nationwide study to describe the clinical impact of surgery on the OS of patients with intestinal NHL. A prospective randomized study evaluating strategies to improve the survival of intestinal NHL patients is needed.

## Introduction

Approximately 30% of malignant lymphomas occur in sites other than the lymph nodes, spleen, or bone marrow [1]. The most common site of extranodal lymphoma is the gastrointestinal tract, and most gastrointestinal tract lymphomas are non-Hodgkin lymphoma (NHL) [2]. In the context of gastric lymphoma treatment, it is essential to recognize extranodal marginal zone B-cell lymphoma of mucosal-associated lymphoid tissue (MALT lymphoma) as a distinct clinical entity. The regression of gastric MALT lymphoma is clearly linked to the eradication of *Helicobacter pylori* (*H. pylori*). Consequently, antibiotic treatment to eliminate *H. pylori* remains the standard initial therapy for gastric MALT lymphoma [3]. Previous studies have demonstrated that the efficacy of conservative management is the same as that of surgery in patients with localized gastric lymphoma [4]. Nonsurgical methods, including *H. pylori* eradication, chemotherapy, and radiation, have been increasingly used in the treatment of gastrointestinal NHL in the past decade [5]. Surgery is no longer recommended for patients with gastric lymphoma because it is not superior to irradiation and can lead to local complications and poor quality of life [6]. Moreover, for patients with intestinal diffuse large B-cell lymphoma, surgical resection followed by chemotherapy might be a more effective treatment strategy than chemotherapy alone [7].

Because intestinal NHL is rare, the role of surgery in its treatment requires reevaluation. Owing to the heterogeneity associated with the anatomical and histological distribution of intestinal NHL, studies focusing on intestinal NHL in nationwide patient samples using the latest 5th edition of the World Health Organization (WHO) classification of NHLs are needed to understand this disease entity. Therefore, we analyzed the impact of surgical resection on survival outcomes in patients with intestinal NHL who underwent chemotherapy.

## Methods

### Sources of data

Our analysis utilized the National Health Insurance Service (NHIS) claim database, a definitive resource in the Republic of Korea. The NHIS database provides extensive information including patient demographics (sex and date of birth), disease diagnoses, detailed medical utilization records, prescription history, and health examination results. This comprehensive coverage of the entire population’s health and medical services ensures robust generalizability for our findings. Given its high representativeness, we secured approval from the NHIS data access committee to use this valuable de-identified information. This study was reviewed and approved by the Institutional Review Board (IRB) of Hanyang University Guri Hospital (Approval No. 2024-04-007), which was conducted in accordance with the Declaration of Helsinki. This study complied with STROBE (Strengthening the Reporting of Observational Studies in Epidemiology) guidelines. The committee waived the requirement for written consent because the data in this national dataset were anonymized for research purposes.

### Patient identification

NHL patients were retrospectively identified using the Korean Classification of Diseases (KCD), which aligns with International Classification of Diseases (ICD)-10. Since 2002, specific KCD codes (C82–C86) have been consistently used for NHL. Subtype definitions were based on the following codes: diffuse large B-cell lymphoma (DLBCL) (C83.3, C83.8, C83.9, C85.1, C85.7), follicular lymphoma (FL) (C82, C82.0–C82.5, C82.7, C82.9), mantle-cell lymphoma (MCL) (C83.1), Burkitt Lymphoma (C83.7), lymphoplasmacytic lymphoma (LPL) (C83.0), angioimmunoblastic T-cell lymphoma (AITL) (C86.5), peripheral T-cell lymphoma (PTCL) (C84.4), enteropathy-associated T-cell lymphoma (MEITL) (C86.2), NK/T-cell lymphoma (NKTCL) (C84.5, C84.9, C86.0, C86.4), anaplastic large-cell lymphoma (ALCL) (C84.6, C84.7), and other/unspecified NHL (C85.9). The records of a total of 74,527 patients with NHL diagnostic codes were collected from January 2002 to December 2021, without considering their disease sites. We subsequently excluded patients whose disease site was not in the intestinal area distinguished by the code of endoscopy, the codes of lymphoma biopsy, or the codes of surgery type (*n* = 52,591). We excluded gastric lymphoma patients who had codes for stomach surgery or esophagogastroduodenoscopy to maximize the accuracy of the intestinal NHL. The codes used for endoscopy were as follows: E7611(esophagogastroduodenoscopy), EZ937 (capsule endoscopy), E7660 (colonoscopy), E7670 (rectoscopy), E7680 (sigmoidoscopy), E7690 (peritoneoscopy), E7691 (retroperitoneoscopy), and E7700 (culdoscopy). The codes used for lymphoma biopsy were C8031 (bone marrow aspiration), C8531 (skin incisional biopsy), C8532 (lymph node: axillary, orbit, nasal cavity, ear, oral cavity, face, external genitalia; incisional biopsy), C8533 (operative biopsy, intrathoraxic), C8534 (operative biopsy, intraabdominal), C8535 (muscle and soft tissue incisional biopsy), P2102 (superficial cervical lymph node excision), P2103 (deep cervical lymph node excision), P2121 (axillary lymph node excision), P2122 (axillary lymph node dissection), P2141 (inguinal lymph node excision), and P2142 (inguinal lymph node dissection). The codes of surgery were resection of the small intestine (Q2650, Q2651), colectomy (QA671, Q2671, Q1261, Q1262, QA672, Q2672), segmental resection of the colon (QA673 Q2673), appendectomy (Q2861, Q2862, Q2863), resection of the rectal tumor (Q2890, Q2891, Q2892, Q2893), anterior resection (Q2921, QA921, Q2927), low anterior resection (Q2922, QA922), ultralow anterior resection (Q2928, QA928), abdominal peritoneal resection (Q2923, QA923), abdominal pull-through operation (Q2924, QA924), and total coloprotectomy (QA925, Q2925, QA926, Q2926). The codes for surgeries with lymph node dissection (LND) were Q1261 Q2651 QA671 QA672 QA673 QA921 QA922 QA923 QA924 QA928 QA925 QA926 Q2927, and the codes for surgeries without LND were Q1262 Q2650 Q2671 Q2672 Q2673 Q2890 Q2891 Q2892 Q2893 Q2921 Q2922 Q2923 Q2924 Q2925 Q2926 Q2928. The codes for colon radical resection were Q1261, Q1262, Q2671, Q2672, QA671, QA672, Q2921, Q2923, Q2924, Q2926, Q2927, Q2928, QA921, QA922, QA923, QA925, QA926, QA928, and colon segmental resection were Q2673, QA673, Q2890, Q2891, Q2892, and Q2893. In the second step, patients who were in the one-year wash-out period were excluded (*n* = 800). In the third step, patients who were younger than 18 years (*n* = 276) were excluded. Finally, we excluded patients who died within 3 months after the diagnosis of lymphoma (*n* = 1,748), patients who underwent open and closed surgery (*n* = 248), and patients who did not receive any chemotherapy (*n* = 6,817). In conclusion, a total of 12,047 patients were followed from the time of intestinal NHL diagnosis until either the end of the study period on December 31, 2021, or the date of death (Supplementary Figure 1). Since the role of surgery was evaluated, all included patients were divided into two groups (nonsurgery and surgery groups) to investigate the relationship between surgical intervention and survival outcomes.

### Endpoint and variables of the study

The primary endpoint was overall survival (OS), which was measured from the first date of diagnosis to the date of death from any cause; patients were censored at the last follow-up date. Patient characteristics included sex, age at diagnosis, census region, type of histology, Charlson Comorbidity Index (CCI), site of involvement, and type of chemotherapy. The chemotherapeutic drugs used were cyclophosphamide, doxorubicin, vincristine, rituximab, etoposide, cisplatin, carboplatin, cytarabine, methotrexate, ifosfamide, l-asparaginase, ibrutinib, bendamustine, gemcitabine, pembrolizumab, nivolumab, lenalidomide, and oxaliplatin. The CCI was calculated on the basis of the presence of the relevant diagnostic disease that occurred within 1 year prior to the diagnosis of intestinal NHL. To maximize the accuracy of coding of intestinal NHL, we used the cancer registration code V193 with the KCD code of NHL.

### Statistical analysis

In this study, descriptive statistics were used to summarize the characteristics of patients with intestinal NHL (Table 1). The baseline characteristics of the patients are presented as means ± standard deviations for continuous variables and as frequencies and percentages for categorical variables. A Cox proportional hazards model was employed for the analysis. The results are reported as hazard ratios (HRs) along with their corresponding 95% confidence intervals (CIs). Variables that exhibited significance with a *p* value <0.05 in the univariable analysis were included in the multivariable analysis. A forest plot was used to present HRs and 95% CIs for various risk factors associated with overall survival. SAS Enterprise Guide Software 7.1 (SAS Institute Inc.) and R version 4.0.3 (R Foundation for Statistical Computing) were used for all the statistical analyses. Two-tailed tests were used for statistical tests, and a significance level of 0.05 was applied.

**Table 1.** Baseline characteristics of the patients.

	Nonsurgery (*n* = 8481, 70.4%)	Surgery (*n* = 3566, 29.6%)	Total (*n* = 12047, 100.0%)	*p* value
**Age (years)**	60.23 ± 13.89	57.8 ± 14.39	59.51 ± 14.08	<0.001
≤60	3945(46.5%)	1910(53.6%)	5855(48.6%)	
>60	4536(53.5%)	1656(46.4%)	6192(51.4%)	
**Sex** – Male	5120(60.4%)	2323(65.1%)	7443(61.8%)	<0.001
Female	3361(39.6%)	1243(34.9%)	4604(38.2%)	
**Histological type**				0.033
**B-cell**	7190(84.8%)	3080(86.4%)	10270(85.3%)	
DLBCL	6245(73.6%)	2838(79.6%)	9083(75.4%)	
FL	337(4.0%)	66(1.9%)	403(3.4%)	
MCL	292(3.4%)	36(1.0%)	328(2.7%)	
Burkitt lymphoma	153(1.8%)	85(2.4%)	238(2.0%)	
LPL	163(1.9%)	55(1.5%)	218(1.8%)	
**T-cell**	703(8.3%)	247(6.9%)	950(7.9%)	
AITL	176(2.1%)	14(0.4%)	190(1.6%)	
PTCL	332(3.9%)	118(3.3%)	450(3.7%)	
MEITL	19(0.2%)	59(1.7%)	78(0.7%)	
NKTCL	106(1.3%)	44(1.2%)	150(1.3%)	
ALCL	70(0.8%)	12(0.3%)	82(0.7%)	
**Others**	588(6.9%)	239(6.7%)	827(6.9%)	
**Charlson comorbidity index**	5.08 ± 2.89	4.47 ± 2.52	4.9 ± 2.8	<0.001
1–3	3080(36.3%)	1580(44.3%)	4660(38.7%)	<0.001
4–6	3400(40.1%)	1400(39.3%)	4800(39.8%)	
>7	2001(23.6%)	586(16.4%)	2587(21.5%)	
Myocardial infarction	163(1.9%)	59(1.7%)	222(1.8%)	0.319
Congestive heart failure	561(6.6%)	167(4.7%)	728(6.0%)	<0.001
Peripheral vascular disease	1121(13.2%)	407(11.4%)	1528(12.7%)	0.007
Cerebrovascular disease	961(11.3%)	287(8.1%)	1248(10.4%)	<0.001
Dementia	266(3.1%)	76(2.1%)	342(2.8%)	0.002
Chronic pulmonary disease	3105(36.6%)	1132(31.7%)	4237(35.2%)	<0.001
Rheumatologic disease	555(6.5%)	131(3.7%)	686(5.7%)	<0.001
Peptic ulcer disease	4108(48.4%)	1675(47.0%)	5783(48.0%)	0.141
Mild liver disease	3487(41.1%)	1341(37.6%)	4828(40.1%)	<0.001
Diabetes without chronic complication	2482(29.3%)	933(26.2%)	3415(28.4%)	0.001
Diabetes with chronic complication	740(8.7%)	234(6.6%)	974(8.1%)	<0.001
Hemiplegia or paraplegia	96(1.1%)	32(0.9%)	128(1.1%)	0.252
Renal disease	319(3.8%)	99(2.8%)	418(3.5%)	0.007
Any malignancy including leukemia and lymphoma	8348(98.4%)	3508(98.4%)	11856(98.4%)	0.815
Moderate or severe liver disease	117(1.4%)	21(0.6%)	138(1.2%)	<0.001
Metastatic solid tumor	1109(13.1%)	301(8.4%)	1410(11.7%)	<0.001
Acquired immune deficiency syndrome/human immunodeficiency virus	45(0.5%)	17(0.5%)	62(0.5%)	0.706
**Census region**				0.994
Referral center	8193(96.6%)	3445(96.6%)	11638(96.6%)	
Secondary care center	288(3.4%)	121(3.4%)	409(3.4%)	
**Intestinal involve site**				<0.001
Small bowel	2(0.0%)	923(25.9%)	925(7.7%)	
Colon	7783(91.8%)	2402(67.4%)	10185(84.5%)	
Rectum	641(7.6%)	241(6.8%)	882(7.3%)	
Others	55(0.7%)	–	55(0.5%)	
**CTx. regimen**				<0.001
CHOP	1579(18.6%)	601(16.9%)	2180(18.1%)	
R-CHOP	6048(71.3%)	2656(74.5%)	8704(72.3%)	
ICE	112(1.3%)	43(1.2%)	155(1.3%)	
GDP	35(0.4%)	10(0.3%)	45(0.4%)	
MTX based	339(4.0%)	68(1.9%)	407(3.4%)	
ESHAP	35(0.4%)	10(0.3%)	45(0.4%)	
R square (Lenalidomide)	14(0.2%)	5(0.1%)	19(0.2%)	
BR	115(1.4%)	26(0.7%)	141(1.2%)	
Immunotherapy	26(0.3%)	7(0.2%)	33(0.3%)	
Ibrutinib	73(0.9%)	7(0.2%)	80(0.7%)	
Others	105(1.2%)	133(3.7%)	238(2.0%)	

### Propensity score matching analysis

Propensity score matching (PSM) analysis was performed to further control confusion, prevent bias, and ensure the reliability of the data. We decided to add variables (sex, age, histological type, CCI score, site of involvement, and type of chemotherapy) to the propensity score model. After propensity score matching (matching for age, sex, chemotherapy regimen, and CCI) at a 1:1 ratio, the matched surgery and nonsurgery groups had the same number of patients. After 1:1 matching on the basis of sex, age, chemotherapy regimen, and CCI score, we compared the outcomes between the surgery and nonsurgery groups. A multivariable Cox proportional hazards model was then used to analyze these groups, adjusting for variables such as sex, age, histological type, CCI score, site of involvement, and type of chemotherapy.

## Results

### Study population

Among the 12,047 patients with intestinal NHL, 3,566 (29.6%) were included in the surgery group and 8,481 (70.4%) were included in the nonsurgery group (Table 1). The mean age at diagnosis was 59 years in intestinal NHL patients. The study cohort comprised more male patients (*n* = 7,443, 61.8%) than female patients. Notably, male patients constituted a significantly higher proportion of the surgery group (65.1%) compared to the nonsurgery group (60.4%; *p* < 0.001). The majority of the patients, 85.3% (*n* = 10,270), were diagnosed with B-cell lymphoma, and 7.9% (*n* = 950) had T-cell lymphoma. DLBCL (*n* = 9,083, 75.4%) was the most frequently diagnosed B-cell lymphoma, followed by FL (*n* = 403, 3.4%), MCL (*n* = 328, 2.7%), Burkitt lymphoma (*n* = 238, 2.0%), and LPL (*n* = 218, 1.8%). The most common T-cell lymphoma was PTCL (*n* = 450, 3.7%), followed by AITL (*n* = 190, 1.6%), NKTCL (*n* = 150, 1.3%), ALCL (*n* = 82, 0.7%), and MEITL (*n* = 78, 0.7%). Most patients (96.6%; *n* = 11,638) were treated in a tertiary referral center. The most common chemotherapy regimen was R-CHOP (rituximab, cyclophosphamide, doxorubicin, vincristine, and prednisone) (72.3%), followed by CHOP (cyclophosphamide, doxorubicin, vincristine, and prednisone) (18.1%). The CCI scores were slightly higher in the nonsurgery group than in the surgery group (mean CCI scores, 5.08 vs. 4.47; *p* < .001).

### Factors associated with overall survival in patients with intestinal NHL

**Figure 1.**
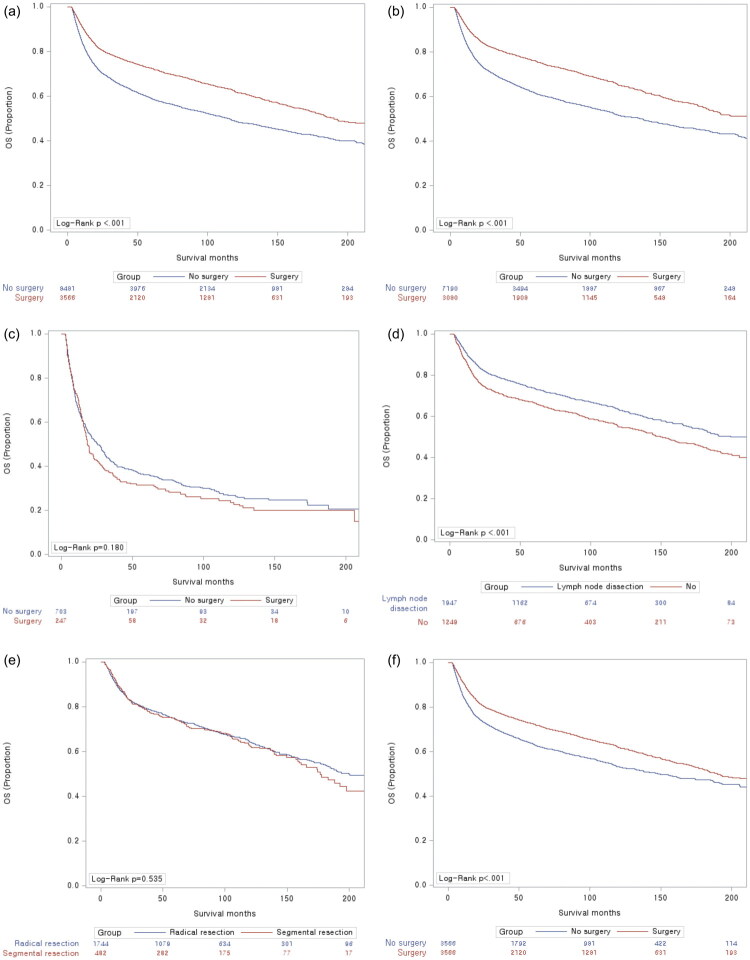
Kaplan–Meier curves for overall survival (A) intestinal NHL (B) B-cell lymphoma (C) T-cell lymphoma (D) OS according to lymph node dissection (E) OS according to type of resection (F) Propensity score-matching (PSM) for OS.

According to the univariate analysis, the factors associated with overall survival in patients with intestinal NHL were male sex (*p* < .001), older age (>60 years) (*p* < .001), histologic type (T cell, others) (*p* < .001), site of involvement (*p* = .001), chemotherapy regimen (*p* < .001), and CCI score (*p* < .001) (Table 2). Before adjusting for the corresponding variables that affected OS in patients with intestinal NHL, multivariate analysis revealed that surgery (HR = 0.669, 95% CI = 0.629–0.713, *p* < .001) not only prolonged OS but also predicted a good prognosis. After adjustment for age, sex, histological type, CCI score, chemotherapy regimen and tumor site, multivariate analysis revealed that surgery (HR = 0.645, 95% CI = 0.598–0.695, *p* < .001) not only prolonged OS but also predicted a good prognosis (Table 3; Figure 1A). However, the benefits of surgery for OS were significant only in B-cell lymphoma patients (*p* < .001) and not in T-cell lymphoma patients (*p* = 0.180) (Figure 1B,C). The Kaplan–Meier curves revealed that the median OS was longer in patients who underwent LND than in patients who did not undergo LND (10-year OS with LND 63.17% vs. surgery without LND 54.78%, *p* < 0.001) (Figure 1D). In patients who underwent colon segmental resection, the median OS was not inferior to that in patients who underwent colon radical resection (10-year OS of 64.18% for colon radical resection vs. 62.41% for colon segmental resection, *p*=.535) (Figure 1E).

**Table 2.** Univariate analysis of Cox proportional hazard ratios for OS.

	OS	*p* value
	HR (95% CI)	
**Age (years)**	1.038 (1.036–1.040)	<0.001
**Sex** – Male	1 (Reference)	<0.001
Female	0.866 (0.818–0.917)	
**Histologic**		<0.001
B-cell	1 (Reference)	
T-cell	2.481 (2.283–2.696)	
Others	1.306 (1.184–1.440)	
**Histologic**		<0.001
DLBCL	1 (Reference)	
FL	0.672 (0.555–0.814)	
MCL	1.352 (1.153–1.586)	
Burkitt lymphoma	1.102 (0.896–1.356)	
LPL	1.795 (1.512–2.132)	
AITL	2.027 (1.674–2.455)	
PTCL	2.769 (2.472–3.101)	
MEITL	4.455 (3.484–5.698)	
NKTCL	2.399 (1.979–2.908)	
ALCL	1.520 (1.113–2.076)	
Others	1.324 (1.200–1.461)	
**Charlson comorbidity index**	1.086 (1.077–1.096)	<0.001
**Census region**		0.477
Referral center	1 (Reference)	
Secondary care center	1.055 (0.911–1.222)	
**Site of involvement**		0.001
Small bowel	1 (Reference)	
Colon	0.946 (0.856–1.045)	
Rectum	0.798 (0.692–0.919)	
Others	0.435 (0.239–0.791)	
**CTx regimen**		<0.001
CHOP	1 (Reference)	
R-CHOP	0.582 (0.545–0.621)	
ICE	1.222 (1.009–1.478)	
GDP	1.435 (1.039–1.981)	
MTX based	1.163 (1.022–1.323)	
ESHAP	0.696 (0.461–1.051)	
R square (Lenalidomide)	0.826 (0.468–1.458)	
BR	0.177 (0.104–0.299)	
Immunotherapy	0.900 (0.572–1.416)	
Ibrutinib	0.528 (0.361–0.773)	
Others	0.966 (0.814–1.145)	

**Table 3.** Multivariate analysis of OS in the surgery and nonsurgery groups.

	OS	*P* value	OS	*P* value
	HR (95% CI)		Adjusted HR^a^ (95% CI)	
No surgery	1 (Reference)	<0.001	1 (Reference)	<0.001
Surgery	0.669 (0.629–0.713)		0.645 (0.598–0.695)	

^a^ Adjust model of P value adjusted for age, sex, histologic, Charlson comorbidity index, CTx. Regimen and tumor site.

### Overall survival after PSM analysis

Among the groups matched for PSM analysis, 3,566 patients who underwent surgery were matched with 3,566 patients who did not. Before adjusting for the corresponding variables that affected OS in patients with intestinal NHL, multivariate analysis revealed that surgery (HR = 0.761, 95% CI = 0.706–0.820, *p* < .001) not only prolonged OS but also predicted a good prognosis. After adjustment for age, sex, histological type, CCI score, chemotherapy regimen and tumor site, multivariate analysis revealed that surgery (HR = 0.656, 95% CI = 0.602–0.714, *p* < .001) not only prolonged OS but also predicted a good prognosis (Table 4; Figure 1F).

**Table 4.** Multivariate analysis of OS in the surgery and nonsurgery groups after matching.

	OS	*P* value	OS	*P* value
	HR (95% CI)		Adjusted HR^a^ (95% CI)	
No surgery	1 (Reference)	<0.001	1 (Reference)	<0.001
Surgery	0.761 (0.706–0.820)		0.656 (0.602–0.714)	

After propensity score-matching (matching for age, sex, regimen, and Charlson Comorbidity Index) with 1:1 ratio, there were both 3566 patients in the matched surgery and nonsurgery groups, respectively.

^a^ Adjust model of *P* values adjusted for age, sex, histologic, Charlson comorbidity index, CTx. regimen and tumor site.

### Subgroup analyses for overall survival in patients with intestinal NHL

Subgroup analyses were performed for surgery, age, sex, histological type, CCI score, site of lymphoma involvement, and chemotherapy regimen (Supplementary Figure 2). Subgroup analysis revealed that age older than 60 years (HR = 1.04, 95% CI = 1.04–1.04, *p* < .001), female sex (HR = 0.84, 95% CI = 0.79–0.88, *p* < .001), histological type (FL, HR = 0.76, 95% CI = 0.62–0.93; MCL, HR = 1.20, 95% CI = 1.02–1.42; Burkitt lymphoma, HR = 1.32, 95% CI = 1.07–1.63; LPL, HR = 1.46, 95% CI = 1.22–1.75; PTCL, HR = 1.96, 95% CI = 1.72–2.24; MEITL, HR = 3.41, 95% CI = 2.63–4.42; NKTCL, HR = 1.61, 95% CI = 1.31–1.98), higher CCI scores (HR = 1.05, 95% CI = 1.04–1.06, *p* < .001), involvement of site (colon, HR = 0.71, 95% CI = 0.63–0.79; rectum HR = 0.67, 95% CI = 0.58–0.78), chemotherapy regimen (RCHOP, HR = 0.64, 95% CI = 0.59–0.70; ICE (ifosfamide, carboplatin, etoposide), HR = 1.36, 95% CI = 1.12–1.65; GDP (gemcitabine, dexamethasone, cisplatin), HR = 1.48, 95%=1.07–2.06; methotrexate based chemotherapy, HR = 1.36, 95% CI = 1.19–1.56; BR (bendamustine, rituximab), HR = 0.28, 95% CI = 0.16–0.48; ibrutinib, HR = 0.41, 95% CI = 0.28–0.61) and surgery (HR = 0.65, 95% CI = 0.60–0.70, *p* < .001) prolonged OS and predicted prognosis (Supplementary Figure 2).

### Stratified analysis

Supplementary Figure 3 presents stratified analyses for OS in the surgery group and nonsurgery group according to age, sex, histological type, CCI score, site of lymphoma involvement, and chemotherapy regimen. Although no significant interaction was found for age, sex, histological type, or CCI score (*P* for interaction > 0.05), the site of lymphoma involvement and chemotherapy regimen had a significant effect on the association. In fact, patients with small bowel intestinal lymphoma could have prolonged OS (*P* for interaction = 0.039) with surgery plus chemotherapy than with chemotherapy alone. Patients who received R-CHOP had prolonged OS (*P* for interaction = 0.021) when they underwent surgery plus chemotherapy than when they received chemotherapy alone.

## Discussion

In this large-scale study of nationwide data of patients with intestinal NHL, we systematically analyzed the clinical impact of surgery on OS. To our knowledge, this is the first Korean population-based nationwide study to describe the clinical impact of surgery on the OS of patients with intestinal NHL.

For intestinal NHL patients older than 18 years who received chemotherapy, surgery was associated with significantly improved OS. Furthermore, the OS of patients who underwent LND during surgery was longer than that of patients who did not undergo LND. Given that there has been little research regarding the association between adequate LND and survival in intestinal NHL patients, this is the first study to reveal the impact of LND on survival in patients with intestinal NHL. This finding suggests that performing LND reduces the tumor burden and improves the prognosis of intestinal NHL patients.

According to previous studies, the indications for surgery were obstruction, perforation, and severe abdominal pain [8]. Although small intestine lesions are difficult to manage and essential diagnostic procedures such as capsule endoscopy are not always feasible, surgery can be a reliable diagnostic technique for small intestine lesions or neoplasms. In this study, compared to the small intestine, a greater proportion of the colon was involved, but considering that patients whose terminal ileum reached the large intestine and was caught by colon surgery codes, the number of patients with involvement of the small bowel may have increased. The potential benefit of adding surgical therapy to non-surgical treatments in advanced gastric lymphoma remains controversial. Historically, some have argued for surgical debulking to reduce tumor burden; however, evidence from controlled clinical trials suggests a more cautious approach. For instance, Avilés et al. demonstrated that in patients with primary gastric DLBCL, chemotherapy alone yielded superior or equivalent results to surgery, while surgical groups experienced higher rates of lethal postoperative complications [9]. Given the increasing use of non-surgical modalities (such as H. pylori eradication, chemotherapy, and radiation therapy), R-CHOP has become the established standard of care for gastric DLBCL, largely replacing radical surgery with organ-preserving strategies. Nevertheless, surgical intervention remains a critical component of multimodal management when clinical complications, such as life-threatening bleeding, obstruction, or perforation, are present [10]. The therapeutic paradigm for gastrointestinal lymphomas has evolved significantly over the past decades. Historically, surgical resection was the cornerstone of treatment, often combined with radiation and chemotherapy to achieve favorable results [11]. However, emerging evidence in the 1990s and early 2000s began to indicate that conservative, non-surgical strategies could achieve equivalent efficacy while preserving organ function [12].

We found that DLBCL was the most common subtype, which is consistent with the literature [13]. Previous data suggest that localized therapies such as surgery cannot prevent systemic relapses and thus are not recommended for patients with localized DLBCL [14,15]. The beneficial effect of surgery was statistically significant only in patients with B-cell lymphomas and not in those with T-cell lymphomas, which is consistent with the literature [16–18]. In general, the prognosis of intestinal T-cell lymphoma is poor [19]. In particular, survival in MEITL patients has remained poor and unchanged over the past 2 decades, considering that more than 30 years have passed since CHOP chemotherapy was developed for T-cell lymphoma treatment, but there is no regimen that can outperform CHOP as the first-line chemotherapy [20–22]. A recent study revealed that the spleen tyrosine kinase (SYK) and programmed death-ligand 1 (PD-L1) expression profiles of MEITL may affect the diagnosis and treatment of this worst type of lymphoma [23].

There are significant racial differences for patients diagnosed with NHLs. Asian patients with NKTCL had the highest age-adjusted incidence rates and the worst survival rate for stage IV DLBCL [24]. In the present study, univariate analysis revealed longer OS in patients under 60 years of age, female patients, patients with indolent lymphoma, patients with lower CCI scores, and those receiving R-CHOP, BR, and ibrutinib chemotherapy.

In T-cell lymphoma patients, the Kaplan–Meier curve was lower in the surgical group than in the nonsurgical group, although the difference was not statistically significant. Our study revealed a relatively lower incidence of intestinal T-cell lymphoma (7.9%), including PTCL, AITL, NKTCL, ALCL, and MEITL, than that reported in previous studies [13,22]. According to the KCD classification, C85.9 patients (non-Hodgkin lymphoma, unspecified) are likely to include patients with T-cell lymphoma. Given that patients with T-cell lymphoma are more likely to have advanced-stage disease and are more refractory to chemotherapy than those with B-cell lymphoma are, the overall survival of T-cell lymphoma patients was inferior to that of B-cell lymphoma patients in this study, which is consistent with previous results [25]. Possible reasons for this observation include postoperative complications, a poor nutritional status, a compromised immune system, and deterioration of general conditions before surgery in patients with T cell lymphoma. Although surgery affects survival in patients with intestinal NHL, intensive postoperative treatment deserves more attention.

The results of this study should be interpreted considering some limitations inherent in the data source. First, the absence of detailed clinical staging and International Prognostic Index (IPI) scores within the NHIS database restricted our ability to perform risk-stratified analyses, which are crucial for lymphoma prognosis. Second, the primary indication for surgery (diagnostic vs. therapeutic) remains undifferentiated within the database, which complicates the accurate assessment of the role of surgical intervention. Furthermore, the NHIS data lack specific records on surgery-related complications and perioperative mortality, precluding a comprehensive evaluation of surgical safety. Third, the potential for selection bias is acknowledged. Although we implemented adjustments, including the exclusion of early mortality (within 3 months of diagnosis), the possibility remains that the surgical group inadvertently comprised patients with inherently better health status, thus potentially overestimating the survival benefit of surgery. Finally, limitations in coding for the anatomical location of intestinal lymphoma may introduce inaccuracies. Specifically, due to a lack of dedicated codes, patients with terminal ileal lymphoma who underwent right hemicolectomy were likely misclassified as having colon-involved lymphoma, which potentially skewed the reported proportion of small intestine lymphoma cases.

Our analysis indicates that surgical intervention, including LND, serves as a significant favorable prognostic factor for OS in patients with intestinal NHL who undergo chemotherapy. While these findings suggest that the role of surgery extends beyond diagnosis, definitive statements regarding its therapeutic contribution are constrained by the observational nature of the data. Beyond survival outcomes, given that the impact on quality of life has become a critical consideration in selecting treatment modalities, future prospective studies are warranted to clarify the optimal role and timing of surgery in the multimodal treatment of intestinal NHL, particularly considering anatomical site, histological subtypes, life quality measurements and potential differences across diverse patient populations. This evidence is necessary to establish optimal, evidence-based treatment guidelines.

## Supplementary Material

Supplemental Material

Supplementary Figure 1.jpeg

Supplementary Figure 2.jpeg

Supplementary Figure 3.jpeg

## Data Availability

The datasets generated during and/or analysed during the current study are available from the corresponding author on reasonable request.

## References

[1] Yang H, Xun Y, Ke C, et al. Extranodal lymphoma: pathogenesis, diagnosis and treatment. Mol Biomed. 2023;4(1):29. doi:10.1186/s43556-023-00141-3. 37718386 PMC10505605

[2] Ponnusamy R, Dasgupta P, Pai A. Intestinal perforation in a case of peripheral T cell lymphoma after initiation of chemotherapy. Kor J Gastroenterol. 2024;84(2):90–94. doi:10.4166/kjg.2024.072. PMC12285490 39176464

[3] Ruskoné-Fourmestraux A, Fischbach W, Aleman BM, et al. Gastric extranodal marginal zone B-cell lymphoma of MALT. Gut. 2011;60(6):747–758. 21317175 10.1136/gut.2010.224949

[4] Raderer M, Chott A, Drach J, et al. Chemotherapy for management of localised high-grade gastric B-cell lymphoma: how much is necessary? Ann Oncol. 2002;13(7):1094–1098. doi:10.1093/annonc/mdf178. 12176789

[5] Nakamura S, Matsumoto T, Iida M, et al. Primary gastrointestinal lymphoma in Japan. Cancer. 2003;97(10):2462–2473. doi:10.1002/cncr.11415. 12733145

[6] Matysiak-Budnik T, Priadko K, Bossard C, et al. Clinical management of patients with gastric MALT lymphoma: a gastroenterologist’s point of view. Cancers (Basel). 2023;15(15):3811. doi:10.3390/cancers15153811. 37568627 PMC10417821

[7] Kim SJ, Kang HJ, Kim JS, et al. Comparison of treatment strategies for patients with intestinal diffuse large B-cell lymphoma: surgical resection followed by chemotherapy versus chemotherapy alone. Blood. 2011;117(6):1958–1965. doi:10.1182/blood-2010-06-288480. 21148334

[8] Tian F-Y, Wang J-X, Huang G, et al. Clinical and endoscopic features of primary small bowel lymphoma: a single-center experience from Mainland China. Front Oncol. 2023;13:1142133. doi:10.3389/fonc.2023.1142133. 37397371 PMC10313208

[9] Avilés A, Nambo MJ, Neri N, et al. The role of surgery in primary gastric lymphoma: results of a controlled clinical trial. Ann Surg. 2004;240(1):44–50. doi:10.1097/01.sla.0000129354.31318.f1. 15213617 PMC1356373

[10] Papaxoinis G, Papageorgiou S, Rontogianni D, et al. Primary gastrointestinal non-Hodgkin’s lymphoma: a clinicopathologic study of 128 cases in Greece. A Hellenic Cooperative Oncology Group study (HeCOG). Leuk Lymphoma. 2006;47(10):2140–2146. doi:10.1080/10428190600709226. 17071488

[11] Fischbach W, Dragosics B, Kolve-Goebeler ME, et al. Primary gastric B-cell lymphoma: results of a prospective multicenter study. The German–Austrian Gastrointestinal Lymphoma Study Group. Gastroenterology. 2000;119(5):1191–1202. doi:10.1053/gast.2000.19579. 11054376

[12] Koch P, Probst A, Berdel WE, et al. Treatment results in localized primary gastric lymphoma: data of patients registered within the German multicenter study (GIT NHL 02/96). J Clin Oncol. 2005;23(28):7050–7059. doi:10.1200/JCO.2005.04.031. 16129843

[13] Kim SJ, Choi CW, Mun YC, et al. Multicenter retrospective analysis of 581 patients with primary intestinal non-Hodgkin lymphoma from the Consortium for Improving Survival of Lymphoma (CISL). BMC Cancer. 2011;11(1):321. doi:10.1186/1471-2407-11-321. 21798075 PMC3160411

[14] Hawkes EA, Barraclough A, Sehn LH. Limited-stage diffuse large B-cell lymphoma. Blood. 2022;139(6):822–834. doi:10.1182/blood.2021013998. 34932795

[15] Matysiak-Budnik T, Fabiani B, Hennequin C, et al. Gastrointestinal lymphomas: French Intergroup clinical practice recommendations for diagnosis, treatment and follow-up (SNFGE, FFCD, GERCOR, UNICANCER, SFCD, SFED, SFRO, SFH). Dig Liver Dis. 2018;50(2):124–131. doi:10.1016/j.dld.2017.12.006. 29301732

[16] Zhang C, Zhang X, Liu Z, et al. The impact of surgery on long-term survival of patients with primary intestinal non-Hodgkin lymphomas based on SEER database. Sci Rep. 2021;11(1):23047. doi:10.1038/s41598-021-02597-1. 34845308 PMC8630038

[17] Wang M, Ma S, Shi W, et al. Surgery shows survival benefit in patients with primary intestinal diffuse large B-cell lymphoma: a population-based study. Cancer Med. 2021;10(10):3474–3485. doi:10.1002/cam4.3882. 33931950 PMC8124121

[18] Chen X, Wang J, Liu Y, et al. Primary intestinal diffuse large B-cell lymphoma: novel insights and clinical perception. Front Oncol. 2024;14:1404298. doi:10.3389/fonc.2024.1404298. 39211552 PMC11357906

[19] Shirwaikar Thomas A, Schwartz M, Quigley E. Gastrointestinal lymphoma: the new mimic. BMJ Open Gastroenterol. 2019;6(1):e000320. doi:10.1136/bmjgast-2019-000320. PMC6782046 31645987

[20] Hujoel IA, Hujoel MLA. The rising incidence and poor outcomes of enteropathy-associated T-cell lymphoma. Am J Gastroenterol. 2024;119(7):1412–1416. doi:10.14309/ajg.0000000000002666. 38235779

[21] Sieniawski M, Angamuthu N, Boyd K, et al. Evaluation of enteropathy-associated T-cell lymphoma comparing standard therapies with a novel regimen including autologous stem cell transplantation. Blood. 2010;115(18):3664–3670. doi:10.1182/blood-2009-07-231324. 20197551

[22] Al Somali Z, Hamadani M, Kharfan-Dabaja M, et al. Enteropathy-associated T cell lymphoma. Curr Hematol Malig Rep. 2021;16(2):140–147. doi:10.1007/s11899-021-00634-4. 34009525

[23] Guo N, Zhou C, Wang Y, et al. Primary intestinal T-cell and natural killer-cell lymphomas: Clinicopathologic and prognostic features of 79 cases in South China. Am J Clin Pathol. 2025;163(1):121–133. doi:10.1093/ajcp/aqae102. PMC11775117 39121027

[24] Kirtane K, Lee SJ. Racial and ethnic disparities in hematologic malignancies. Blood. 2017;130(15):1699–1705. doi:10.1182/blood-2017-04-778225. 28724539 PMC5639484

[25] Hong YW, Kuo IM, Liu YY, et al. The role of surgical management in primary small bowel lymphoma: a single-center experience. Eur J Surg Oncol. 2017;43(10):1886–1893. doi:10.1016/j.ejso.2017.06.016. 28751057

