# Supplementary material for: The surgery for the patients with intestinal non‑Hodgkin lymphomas: a nationwide study
Source: Ann Med. 2026 Feb 24;58(1):2634447. doi: 10.1080/07853890.2026.2634447 (PMC12934337; doi:10.1080/07853890.2026.2634447)
Supplement: Supplemental Material [file IANN_A_2634447_SM4393.docx]

Supplementary Figure1. Flowchart of patients selection and study design.

Supplementary Figure 2. Forest plot of subgroup analyses for overall survival.

Supplementary Figure 3. Stratified subgroup analyses of overall survival between the surgery and no-surgery groups.
