# Supplementary figures and images for: The surgery for the patients with intestinal non‑Hodgkin lymphomas: a nationwide study
Source: Ann Med. 2026 Feb 24;58(1):2634447. doi: 10.1080/07853890.2026.2634447 (PMC12934337; doi:10.1080/07853890.2026.2634447)

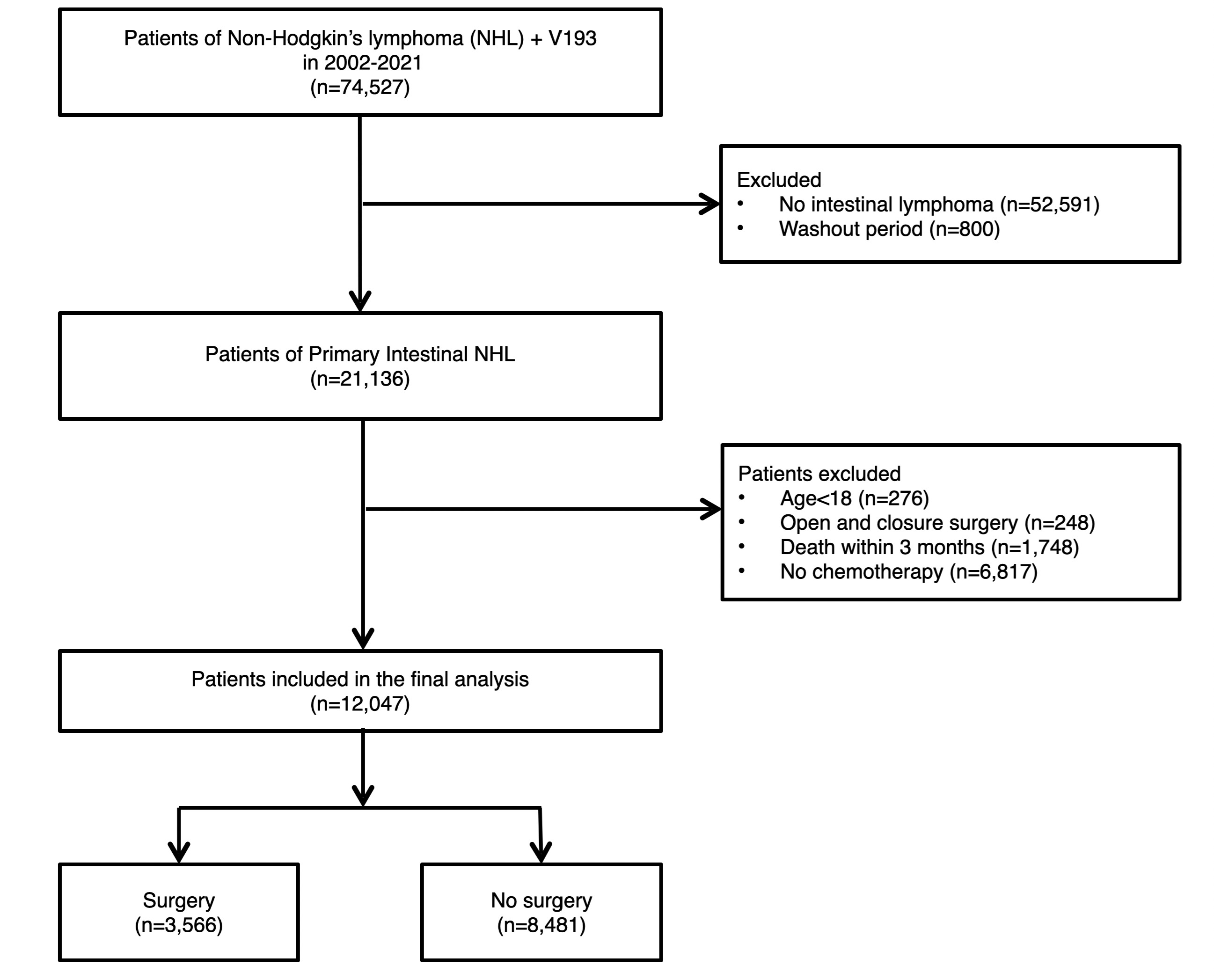

Supplement: Supplementary Figure 1.jpeg [file IANN_A_2634447_SM7618.jpeg]

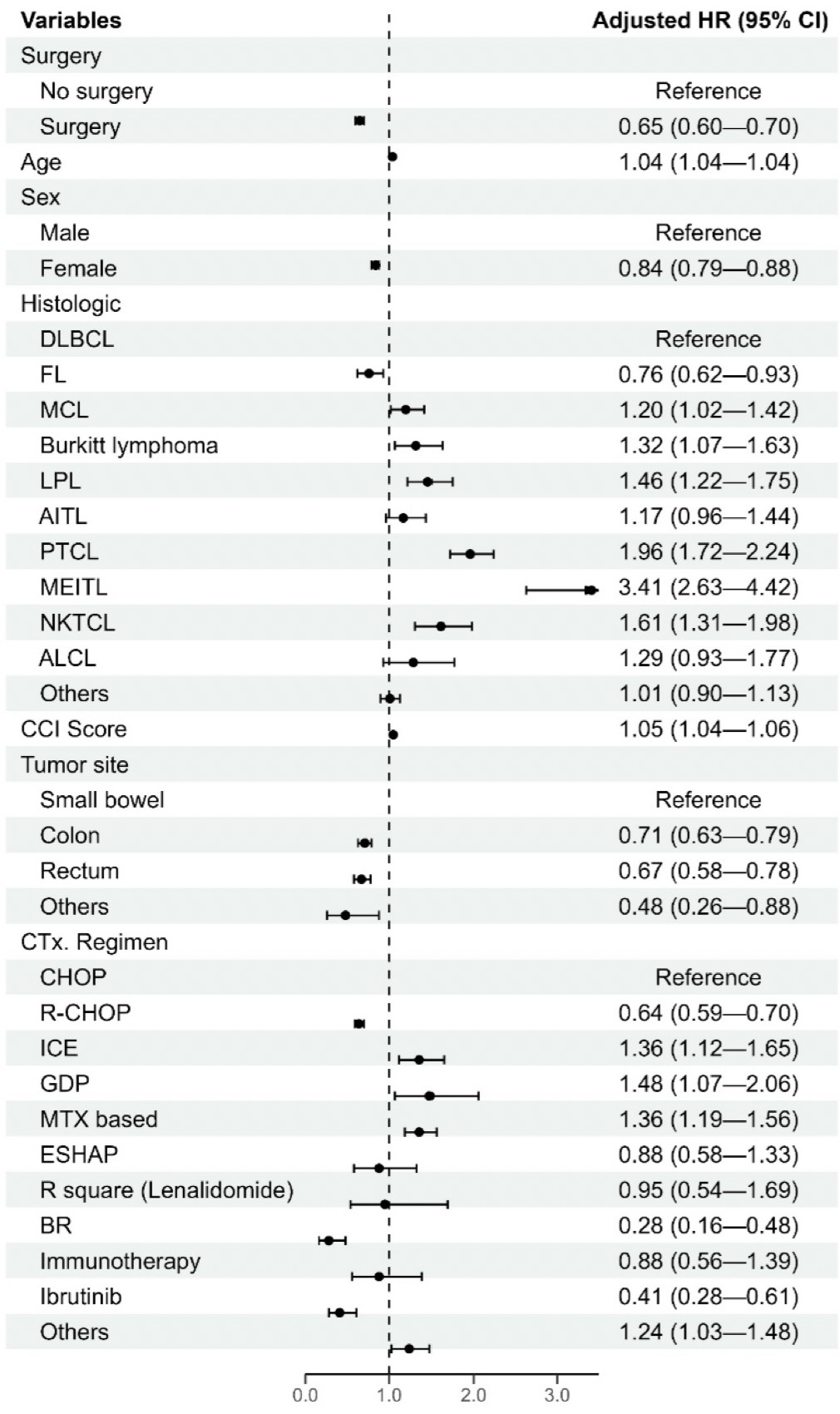

Supplement: Supplementary Figure 2.jpeg [file IANN_A_2634447_SM7617.jpeg]

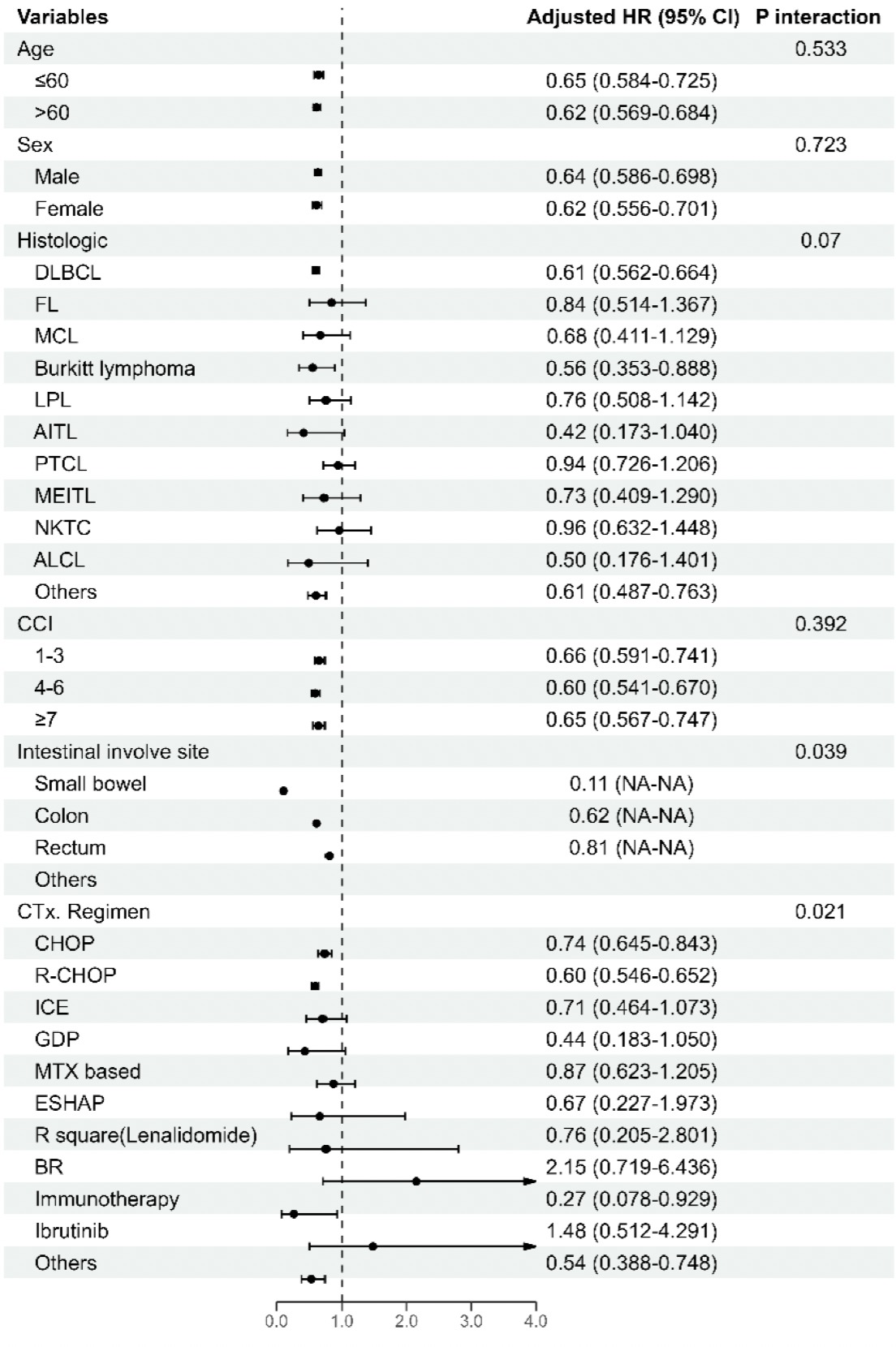

Supplement: Supplementary Figure 3.jpeg [file IANN_A_2634447_SM7616.jpeg]
